# Assessing the Capability of Chemical Ameliorants to Reduce the Bioavailability of Heavy Metals in Bulk Fly Ash Contaminated Soil

**DOI:** 10.3390/molecules26227019

**Published:** 2021-11-20

**Authors:** Joy Kumar Mandal, Siddhartha Mukherjee, Niharendu Saha, Nibedan Halder, Tufleuddin Biswas, Sanjoy Chakraborty, Sabry Hassan, Mohamed M. Hassan, Ali A. Abo-Shosha, Akbar Hossain

**Affiliations:** 1 Department of Agricultural Chemistry and Soil Science, Faculty of Agriculture, Bidhan Chandra Krishi Viswavidyalaya, Mohanpur, Nadia 741252, India; joykr.mandal@gmail.com (J.K.M.); mukherjee.siddhartha1611@gmail.com (S.M.); nihar_bckv@rediffmail.com (N.S.); haldernibedan@gmail.com (N.H.); 2 M.S. Swaminathan School of Agriculture, Centurion University of Technology and Management, Odisha 761211, India; tufleuddin.biswas@cutm.ac.in; 3 Environment Division, Haldia Energy Limited, Haldia 721658, India; sanjoy.chakraborty@rp-sg.in; 4 Department of Biology, College of Science, Taif University, Taif 21944, Saudi Arabia; hassan@tu.edu.sa; 5 Genetics Department, Faculty of Agriculture, Kafrelsheikh University, Kafr El-Sheikh 33516, Egypt; a.aboshosha@gmail.com; 6 Department of Agronomy, Bangladesh Wheat and Maize Research Institute, Dinajpur 5200, Bangladesh

**Keywords:** ameliorants, metal bioavailability, environmental risk, biological indicator

## Abstract

In-situ rehabilitation of fly ash at dumping sites has rarely been addressed for crop production due to growth-related constraints, largely of heavy metal (HM) contamination in soils and crops. Current communication deals with a novel approach to identify a suitable management option for rejuvenating the contaminated soils. In this background, a 60-days incubation experiment was conducted with different fly ash-soil mixtures (50 + 50%, A1; 75 + 25%, A2; 100 + 0%, A3) along with four ameliorants, namely, lime (T1), sodium sulphide (T2), di-ammonium phosphate (T3), and humic acid (T4) at 30 ± 2 °C to assess the ability of different fly ash-soil-ameliorant mixtures in reducing bio-availability of HMs. Diethylenetriaminepentaacetic acid (DTPA)-extractable bio-available HM contents for lead (Pb), cadmium (Cd), nickel (Ni), and chromium (Cr) and their respective ratios to total HM contents under the influence of different treatments were estimated at 0, 15, 30, 45, and 60 days of incubation. Further, the eco-toxicological impact of different treatments on soil microbial properties was studied after 60 days of experimentation. A1T1 significantly recorded the lowest bio-availability of HMs (~49–233% lower) followed by A2T1 (~35–133%) among the treatments. The principal component analysis also confirmed the superiority of A1T1 and A2T1 in this regard. Further, A1T1 achieved low contamination factor and ecological risk with substantial microbial biomass carbon load and dehydrogenase activity. Thus, liming to fly ash-soil mixture at 50:50 may be considered as the best management option for ameliorating metal toxicity. This technology may guide thermal power plants to provide the necessary package of practices for the stakeholders to revive their contaminated lands for better environmental sustainability.

## 1. Introduction

Fly ash is the end residue from the combustion of crushed coal in the furnace of thermal power plants and comprises mineral constituents of coal which is not entirely burnt. It contains fine powdery spherical, amorphous ferro-aluminosilicate particles and is globally considered to be a hazardous waste to the ecosystem since, it generally contains various toxic heavy metals such as Pb, Cd, Cr, Ni, Hg, V, B, As, Se [1]. In the process of burning coal, the organic part is eliminated and subsequently, enhancement and accumulation of the heavy metals from coal to ash take place [2]. However, in many countries, this industrial by-product has not been appropriately utilized rather it has been ignored and dumped like a leftover substance. As a consequence, it poses a significant threat to the soil environment, streams as well as groundwater [3,4] and becomes a noxious source of health hazards for humans and animals when reached to the domestic areas [5,6]. Furthermore, the dumping sites do not support sound plant growth because of its nutritional deficiency (generally N and P), low microbial activity, high salt concentration along enhanced heavy metal loads [7,8]. Moreover, disposal of such a high amount of fly ash requires enormous water, energy, and land areas [9]. Though it is not estimated scientists speculate that there are more than 82,200 ha area of only fly ash ponds in India adjacent to the power plant areas [4]. Therefore, appropriate fly ash management would remain a great apprehension of the century.

Many researchers have tried to utilize fly ash in agriculture use [8,10]. However, the major problem related to the application of fly ash in agriculture is heavy metal accumulation in the soil environment. A few attempts in this regard have been initiated by different researchers to reduce metal contaminations in different ways. Ecological engineering by employing phytoremediation and bioremediation is very useful to lessen the hazardous effects of the fly ash dump sites in many studies [11,12]. However, those methods do not address immediate risk minimization rather it takes a certain time, sometimes years to restrict the toxic nature of fly ash. Other than that, very few amendments such as farmyard manure, press mud, compost, and bio-solids have been studied to stabilize fly ash with limited success [13,14]. No other scientific method has been defined to stabilize dumps in this regard.

Chemical immobilization is a remediation technique that decreases the concentration of dissolved contaminants by sorption and/or precipitation in heavy metal contaminated soils. Their applicability is mainly concentrated on reducing the bioavailability, solubility, or extractability of metals. In this context, ameliorants such as lime, sodium sulphide (SS), di-ammonium phosphate (DAP), humic acid have been studied successfully in a vast number of studies [15,16]. However, their applicability to reclaim fly ash in situ at dumping sites to make the sites productive has never been studied. Thus, the cognizance of the applicability of chemical ameliorants in heavy metal contaminated soils led us to this background to test their applicability in our experiment. Fifteen different combinations of fly ash-soil-ameliorant involving 3 fly ash-soil combinations along with 4 different ameliorants (lime, DAP, Na_2_S, and humic acid) were employed in an incubation study to find out the most suitable treatment combination in reducing the bio-availability of heavy metals. Probably, this study will help to develop a scientific method for immobilizing and reducing the bio-availability of heavy metals within the shortest period. The outcome of the study will provide technical know-how to the power plant authorities to manage huge fly ash in the soil environment safely.

## 2. Results

This section is divided into the following sub-headings. It should provide a concise and precise description of the experimental results, their interpretation, as well as the experimental conclusions that can be drawn.

### 2.1. Bio-Availability of Heavy Metals

**Figure 1:**
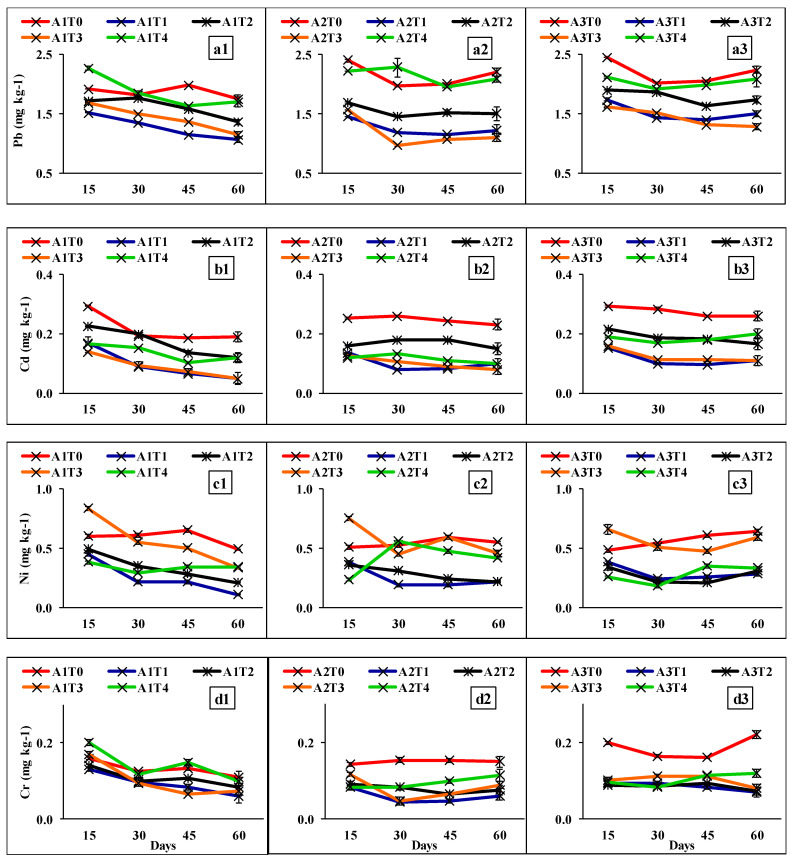
DTPA-extractable of heavy metals ((**a1**–**a3**): Pb, (**b1**–**b3**): Cd, (**c1**–**c3**): Ni, (**d1**–**d3**): Cr; mg kg^−1^) during 60 days of incubation. Error bars indicate the standard error of the mean. A1: fly ash (50%) + soil (50%), A2: fly ash (75%) + soil (25%), A3: fly ash (100%); T0: without ameliorants, T1: lime at 5 Mg ha^−1^, T2: sodium sulfide at 2 Mg ha^−1^, T3: di-ammonium phosphate at 0.5 Mg ha^−1^, T4: humic acid at 4 Mg ha^−1.^.

DTPA-extractable bioavailable fraction of heavy metals was varied across different stages of incubation for various fly ash-soil-ameliorants combinations used in this experiment (Figure 1). The extractable amounts of Pb, Cd, Ni, and Cr significantly differed for different treatment combinations after 60 days of incubation (Figure 1a1–d1) and a noticeable reduction in metal concentration was detected in different combinations.

**Figure 2:**
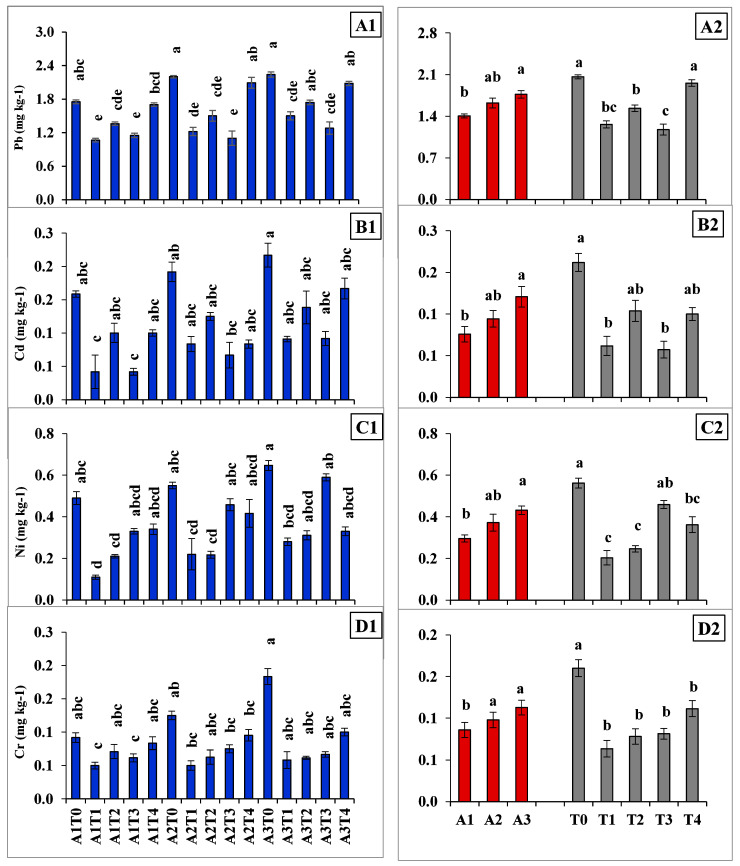
Bioavailability of heavy metals (Pb, (**A1**,**A2**); Cd, (**B1**,**B2**); Ni, (**C1**,**C2**); Cr, (**D1**,**D2**)) after 60-days incubation under different treatment combinations. A1: fly ash (50%) + soil (50%), A2: fly ash (75%) + soil (25%), A3: fly ash (100%); T0: without ameliorants, T1: lime at 5 Mg ha^−1^, T2: sodium sulfide at 2 Mg ha^−1^, T3: di-ammonium phosphate at 0.5 Mg ha^−1^, T4: humic acid at 4 Mg ha^−1^. Values followed by different lowercase letters are significantly different by Tukey’s test (*p* = 0.05) for a heavy metal. Error bar represents the standard error of mean.

A1T1 recorded the lowest value for bio-available Pb (1.07 mg kg^−1^; ~49% lower), Cd (0.05 mg kg^−1^, ~171% lower), Ni (0.11 mg kg^−1^, ~233% lower), and Cr (0.06 mg kg^−1^, ~65% lower) whereas A3T0 recorded the highest (2.24, 0.26, 0.65, 0.22 mg kg^−1^ respectively, for the same; ~29–55% higher) among the treatments. On an average, the amount of DTPA-extractable Pb, Cd, Ni, and Cr was increased with the increased proportion of fly ash in fly ash-soil combinations (A3 > 1.09 A2 > 1.26 A1, A3 > 1.28 A2 > 1.59 A1, A3 > 1.16 A2 > 1.46 A1, and A3 > 1.15 A2 > 1.31 A1 for Pb, Cd, Ni, and Cr respectively) irrespective of ameliorants applied (Figure 2A2–D2). However, the efficacy of ameliorants in reducing the bio-availability of heavy metals was varied significantly within themselves. In this regard, T1 (mean ~1.26, 1.51, 1.83, and 1.65 times lower for Pb, Cd, Ni, and Cr, respectively) retained the lowest amount of bio-available HMs followed by the variant T3 (mean ~1.35, 1.70, 0.80, and 1.24 times lower, respectively, for the same) among all the treatments.

### 2.2. Changes in DTPA-Extractable Heavy Metal to Total Heavy Metal Ratio under Different Fly Ash-Soil-Ameliorant Combinations

**Figure 3:**
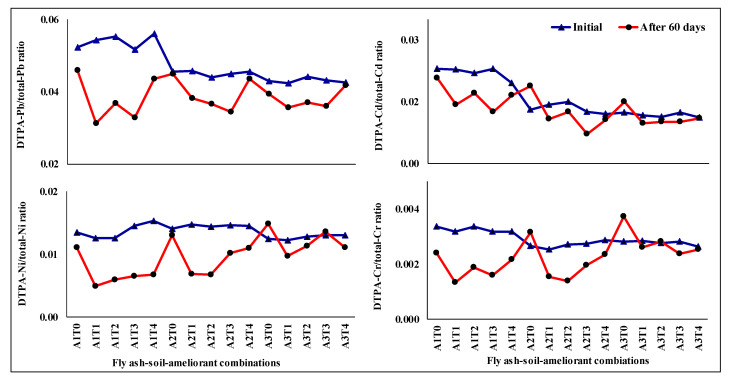
Changes in DTPA extractable heavy metal to the total-heavy metal ratio for Pb, Cd, Ni, and Cr under different fly ash-soil-ameliorant combinations after 60-days incubation. A1: fly ash (50%) + soil (50%), A2: fly ash (75%) + soil (25%), A3: fly ash (100%); T0: without ameliorants, T1: lime at 5 Mg ha^−1^, T2: sodium sulfide at 2 Mg ha^−1^, T3: di-ammonium phosphate at 0.5 Mg ha^−1^, T4: humic acid at 4 Mg ha^−1^.

After 60 days of incubation, the ratio of DTPA-extractable to total heavy metal concentrations for Pb, Cd, Ni, and Cr was varied from its initial across the interventions imposed (Figure 3).

On average, a declining trend was observed in the ratio for all the heavy metals with the application of different ameliorants (mean~0.009, 0.003, 0.004, and 0.001 for Pb, Cd, Ni, and Cr respectively). All the treatments comprised of A1 i.e., fly ash-soil (50% + 50%) combination showed a greater declining trend as compared to A2 (fly ash-soil: 75% + 25%) and A3 (fly ash: 100%), respectively, irrespective of the heavy metals considered (A1 > 2 A2 > 5 A3). Overall, A1T1 (0.01) followed by A1T3 (0.009) recorded the highest decrease whereas A2T0 (−0.001) followed by A3T0 (−0.0006) recorded the lowest among the treatments. In most cases, these ratios for different metals were highly correlated with their respective bioavailable fraction than the total metal load under all the treatments (Table 1).

### 2.3. Efficacy of Different Fly Ash-Soil-Ameliorant Combinations in Minimizing Bio-Availability of Heavy Metals

**Figure 4:**
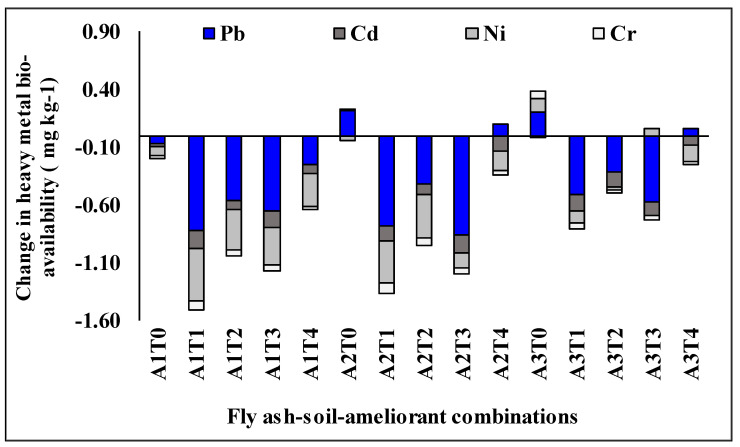
Changes in bioavailability of heavy metals (mg kg^−1^) under different fly ash-soil-ameliorant combinations after 60 days of incubation. A1: fly ash (50%) + soil (50%), A2: fly ash (75%) + soil (25%), A3: fly ash (100%); T0: without ameliorants, T1: lime at 5 Mg ha^−1^, T2: sodium sulfide at 2 Mg ha^−1^, T3: di-ammonium phosphate at 0.5 Mg ha^−1^, T4: humic acid at 4 Mg ha^−1^.

Among different treatment combinations, a greater decrease of Pb and Cd by A2T3; Ni by A1T1, and Cr by A2T1 in the tune of 0.89, 0.15, 0.46, and 0.07 mg kg^−1^ was achieved after 60 days incubation. However, considering the four heavy metals, the total decrease was highest for A1T1 (1.43 mg kg^−1^; ~226% decrease) followed by A2T1 (1.36 mg kg^−1^; ~126% decrease) averagely whereas A3T0 (0.26 mg kg^−1^, mean ~10% increase) recorded the lowest (Figure 4). Overall, among the treatments, the trend in decreasing the bio-availability of heavy metals observed in this experiment was: A1T1 > A2T1 > A2T3 > A1T3 > A2T2 > A1T2 > A3T1 > A3T3 > A3T2 > A1T4 > A3T4 > A2T4 > A1T0 > A2T0 > A3T0.

PCA results showed that ~97% of the total variation in declining the bioavailability of HMs for different fly ash-soil-ameliorant combinations was explained by the first three components (Table 2). Principle component 1 (PC1) explained 73% of the total variance having a significant loading on each of the variables. The highest loading was attained by the A1T1 treatment combination in PC1 (PC1 loading on A1T1: 2.32). The second (PC2) and third principal components (PC3) explained only ~18 and 6% of the total variance respectively and loaded highest on A2T3 and A1T0.

**Figure 5:**
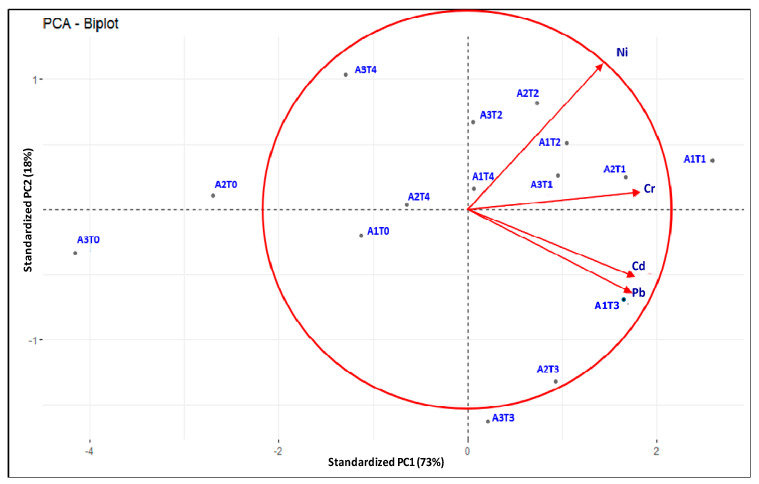
Evaluation of different fly ash-soil-ameliorants combinations in reducing bio-availability of heavy metals through PCA (principal component analysis)-scatterplot. A1: fly ash (50%) + soil (50%), A2: fly ash (75%) + soil (25%), A3: fly ash (100%); T0: without ameliorants, T1: lime at 5 Mg ha^−1^, T2: sodium sulfide at 2 Mg ha^−1^, T3: di-ammonium phosphate at 0.5 Mg ha^−1^, T4: humic acid at 4 Mg ha^−1^.

Using PC1 and PC2, we analyzed PCA biplot (Figure 5), which signifies the performance of the fifteen different treatments combinations (points in the plane) in reducing HMs bioavailability considering four different HMs (arrows/vectors). The vectors begin at the origin (0,0) and extend to coordinates given by the loading vectors. The treatments positioned on the positive *X*-axis i.e., closer to the vectors are the good performing treatment with respect to that variable(s). In this biplot, treatments such as A1T1, A2T1, and A1T3 performed brilliantly concerning all HMs and among those three treatments, the relative performance of A1T1 was better for all four vectors in reducing their bio-availability. On the other hand, the performance of the remaining treatments was relatively poor where A3T0 followed by A2T0 were considered as the poorest. Accordingly, A1T1 was found as the most suitable soil-fly ash-ameliorant combination in this experiment to serve our objectives.

### 2.4. Evaluation of Environmental Risk

**Figure 6:**
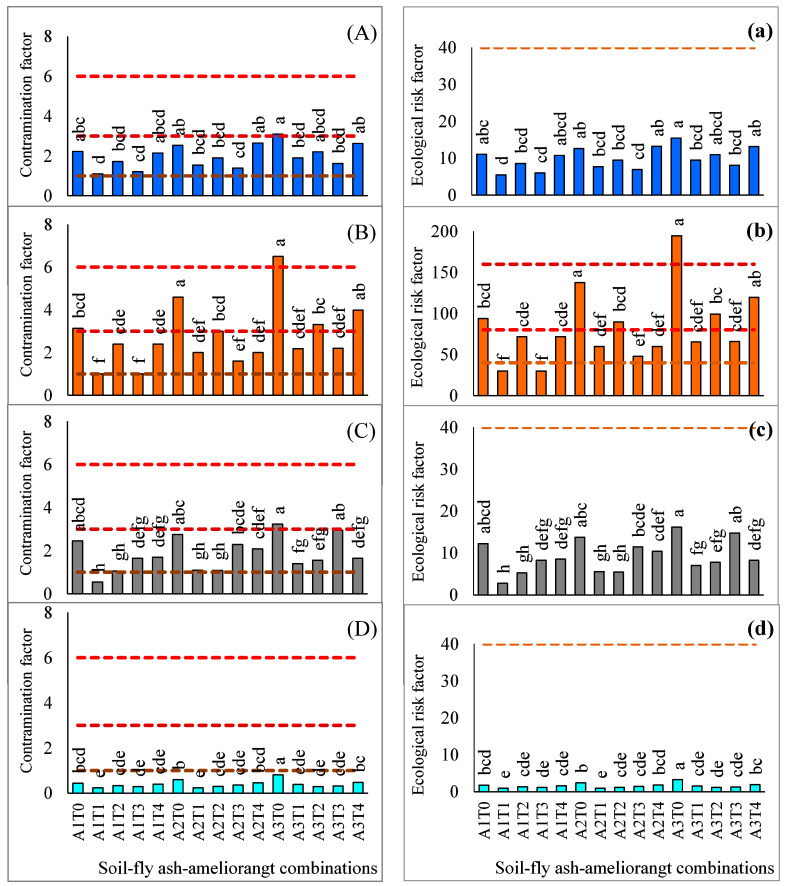
Contamination factor (CF: Pb, (**A**); Cd, (**B**); Ni, (**C**); Cr, (**D**)) and ecological risk factor (ERF: Pb, (**a**); Cd, (**b**); Ni, (**c**); Cr, (**d**)) of different fly ash-soil-ameliorant combinations for different metals. CF < 1: low, 1–3: moderate, 3–6: considerable, >6: very high contamination; ERF < 40: low, 40–80: moderate, 80–160: considerable, >160: very high ecological risk. A1: fly ash (50%) + soil (50%), A2: fly ash (75%) + soil (25%), A3: fly ash (100%); T0: without ameliorants, T1: lime at 5 Mg ha^−1^, T2: sodium sulfide at 2 Mg ha^−1^, T3: di-ammonium phosphate at 0.5 Mg ha^−1^, T4: humic acid at 4 Mg ha^−1^.

The efficacy of different amendments to improve fly ash-soil quality in terms of reduced mental load and its impact on soil ecology was evaluated by using the two most widely used indexes i.e., contamination factor (CF) and ecological risk factor (ERF) for four different HMs (Figure 6).

Values for CF for different metals under the influence of different fly ash-soil-ameliorant combinations significantly varied among themselves. All the treatment combinations exhibited a ‘moderate contamination’ level for Pb (CF: 1–3) excepting the A3T0 combination which showed a ‘considerable contamination’ (CF: 3–6). Pollution load for Cd under the influence of A1T0 and A2T0 showed a ‘considerable contamination’ level whereas the others depicted a ‘moderate contamination’ considering all A1 and A2 combinations. In addition, a ‘considerable contamination’ level for Cd was attained for all the treatments constituted by 100% fly ash in conjunction with different inorganic and organic amendments excepting sole application of fly ash (A3T0) which recorded the ‘high contamination’ level (CF: >6). For Ni, only A1T1 had a ‘low contamination’ level and the others presented mostly of moderate level. A3T0, A3T2, A3T3 had a value between 3 to 6 and categorized ‘considerable contamination’ level. All the treatments showed a ‘low contamination’ level for Cr apart from A3T0 which was categorized under ‘moderate contamination’ level.

Results for ERF showed that all the treatment combinations had a value less than 40 and were classified under ‘low ecological risk’ for Pb, Ni, and Cr. However, the values of ERF for Cd varied considerably. A3T0 showed a ‘high ecological risk’ (ERF: >160) whereas A1T0, A2T0, and all other treatments comprising A3-ameliorant combinations were classified under ‘considerable ecological risk’ (ERF: 80–160) for Cd. A1T1 and A1T3 recorded a value less than 40 and were considered as ‘low ecological risk’ and the remaining treatments presented moderate level-values for the same.

### 2.5. Assessment of Microbial Endpoints under the Influence of Fly Ash-Soil-Ameliorant Combinations

To support the environmental risk factor in the earlier section, two widely used soil health indicators viz. microbial biomass carbon (MBC) and dehydrogenase activity under the influence of different treatment combinations were assessed (Table 3).

Among the base material, MBC load and dehydrogenase activity was highest in A1 followed by A2 and A3 (A1 > 1.8A2 > 8.9A3 and A1 > 1.9A2 > 19.7A3 respectively), irrespective of the ameliorants applied. While different ameliorants were imposed on the base materials, a noticeable increase in MBC was recorded in A1 followed by A2 over sole fly ash as a base material (A3) by 52 and 26 µg g^−1^. However, in all cases, irrespective of the base material, T4 excelled over others. On the whole, A1T4 holds significantly higher MBC followed by A1T1 by ~493% and 391%, respectively. A similar trend was also followed in the case of dehydrogenase activity under different treatment combinations and A1T4 followed by A1T1 recorded the highest values (~9 and 7 µg TPF g^−1^ h^−1^ respectively) over others.

## 3. Materials and Methods

### 3.1. Raw Material

Fly ash was randomly collected from three spots of the fly ash storage facility (ash pond) of Haldia Energy Limited (HEL) power plant, a subsidiary of Calcutta Electric Supply Corporation (CESC, Haldia, India) in 2018. It was located at Baneswarchak, Haldia, East Medinipur, WB, India (22°06’ N 88°10’ E and 6 m above mean sea level). The power plant has a capacity of 2 × 300 MW and the required coal is supplied from Mahanadi Coalfields Ltd. which mainly provides bituminous coal from the mines of Odisha and Jharkhand (www.haldiaenergy.co.in, accessed on 7 April 2021). The collected fly ash was thoroughly mixed to prepare a representative sample and subsequently air-dried, sieved with 2-mm mesh, and characterized for different physical, chemical, and biological parameters following the standard procedures [14].

### 3.2. Experimental Soil

To comprise different fly ash-soil combinations, the required amount of soil was collected from Central Research Farm, Bidhan Chandra Krishi Viswavidyalaya, Gayeshpur (22°58′ N, 88°29′ E and 9.75 m above mean sea level) under the new alluvial zone of West Bengal, India. The zone falls under a sub-tropical, humid climate having an average annual rainfall of approximately 1550 mm and pan evaporation of 1.2 to 5.0 mm day^−1^. The mean annual maximum and minimum temperatures were 36.3 °C and 12.5 °C, respectively [17]. The soil was qualified for the hyperthermic temperature class and classified as Aeric Haplaquept [18] with silty loam texture. Soils were sampled from the surface layer (0–20 cm depth), stored in polyethylene bags and all the samples were bulked together in the laboratory to obtain an “average” sample. Soil material was homogenized, air-dried, and manually crushed. After that, the average sample was passed through a 2-mm sieve and subsequently analyzed for different physical, chemical, and biological properties following the standard procedures as proposed by Page et al. [19]. Some important soil properties have been presented in Table 4.

### 3.3. Experimental Details and Analysis

Three altered combinations of fly ash and soil (fly ash—50, 75, 100%) along with four different ameliorants, namely, lime (T1), sodium sulphide/Na_2_S (T2), di-ammonium phosphate/DAP (T3), and humic acid (T4) were used to comprise different treatment combinations for this experiment (Table 5). All the treatments were laid out in a completely randomized design (CRD) with three replications to assess their ability in reducing the bio-availability of heavy metals in the soil environment.

For the incubation experiment, 1 kg of processed fly ash was taken in each of the forty-five different plastic pots (15 cm diameter). The required amount of soil and ameliorant were added to the pots according to the specific treatment combination and thoroughly mixed. The treatment combinations in different pots were maintained at field capacity (28.2% *w/w*) throughout the incubation period (60 days) by adding the same volume of distilled water lost due to evaporation. The pots were incubated at 30 ± 2 °C in an incubator. Soil from each of the pots was sampled after 15, 30, 45, 60 days and immediately analyzed for bio-availability of different heavy metals (Pb, Cd, Cr, and Ni contents) following the standard procedure as suggested by Lindsay and Norvell [26]. The estimation was carried out using DTPA extraction procedure (0.005 M diethylenetriaminepentaacetic acid (DTPA) + 0.01 M CaCl_2_·2H_2_O + 0.1 M tri-ethanol amine or TEA), buffered at pH 7.3. Subsequently, the concentrations of these elements in the soil extracts were analysed by an atomic absorption spectrophotometer (PerkinElmer PinAAcle^TM^ 900F, Waltham, MA, USA).

To find out the total heavy metal concentrations in different fly ash-soil-ameliorant combinations, samples were digested (wet acid digestion) with concentrated HNO_3_, H_2_SO_4,_ and HClO_4_ (5:1:1) at 80 °C [29] until the solution became transparent. The digested samples were filtered through the Whatman No.42 filter paper and the filtrates were diluted to 50 mL with distilled water. All used reagents were Merck, analytical grade (AR) including standard stock solutions of known concentrations for different heavy metals. The heavy metal (Pb, Cd, Ni, Cr) concentrations of these samples were analyzed by an atomic absorption spectrophotometer. The monochromator was set at the wave-length of 213.9 nm, 228.8 nm, 232.0, and 357.9 for Pb, Cd, Ni, and Cr respectively at the time of estimation and the programming was controlled using Perkin-Elmer Gem software (version 7.2.1).

Biological parameters like microbial biomass carbon (MBC) by chloroform fumigation method [27] and dehydrogenase activity by tri-phenyl formazan (TPF) method [28] were also measured for different fly ash-soil-ameliorant combinations after 60 days of incubation.

### 3.4. Soil and Environment Risk Assessment

#### 3.4.1. Contamination Factor (CF)

The contamination factor is a very popular soil index used to measure levels of metal pollution in soil. This index determines which element represents the highest threat to a soil environment. The factor is defined as the ratio between the metal concentration in the soil (CM) and the reference concentration of that same metal in unpolluted soil (CB) [30]. From the generated CF values soil can be classified as “very low contamination” (CF < 1) to “very high contamination” (CF > 6).

CF = CM ÷ CB(1)

As the reference concentration of heavy metals in unpolluted soil (CB), the DTPA-extractable Pb, Cd, Ni, and Cr concentrations of the experimental soil (before fly ash addition) were considered to compute this factor. The corresponding values were 0.79, 0.05, 0.20, and 0.25 respectively.

#### 3.4.2. Ecological Risk Factor (ERF)

The ecological risk factor (ERF) is a very useful index to assess the ecological risk of an element in the soil and can be obtained using the following Equation:

ERF = TR × CF(2)

where ‘CF’ is the contamination factor and ‘TR’ is the toxic response factor of an element. The toxic response factors for Pb, Cd, Ni, and Cr are 5, 30, 5, and 2 respectively [30]. ERF values are divided into five ranges from “low potential ecological risk” for ERF < 40 to “very high ecological risk” when ERF > 320.

### 3.5. Statistical Analysis

To meet the normality and additivity criteria of the data set obtained from the experiment were log-transformed and tested by the Shapiro–Wilk test [31]. The significant differences among the treatments were tested by analysis of variance (ANOVA) at 5% probability levels (*p* ≤ 0.05) [32] by Tukey’s test using ‘CRAN R’. Further, to know the performance of the treatments on environmental sustainability, data for contamination factors and ecological risk factors were also compared by Tukey’s test. To eliminate the outliers caused by extreme values in the data set for the parameters like bio-availability of HMs at different incubation stages, contamination factors and ecological risk factors for different HMs were log-transformed [33]. A boxplot analysis also confirmed the absence of outliers in the working data set. Lastly, principal component analysis (PCA) and PCA-biplot were performed with the qualified data set, to screen out the best fly ash-soil-ameliorant combination which contributes minimum in total bioavailability of heavy metals using ‘CRAN R’ software (tidy verse package). To understand the relationship between bio-available (at 60 days) to total heavy metal (HM) ratios and different forms of HMs, Pearson’s correlation study was also performed.

## 4. Discussion

The bio-availability of the four HMs was increased along with the increasing proportion of fly ash in fly ash-soil combinations (Figure 2A2–D2). This is mainly because of the increased load of HM concentrations [14,33,34]. However, the variation in such HM contents within different fly ash-soil combinations during incubation (Figure 1) as well as after 60 days of incubation (Figure 2) may be related to the HM reduction capacity of different ameliorants used in this experiment. The decrease in the availability of Pb, Cd, Ni, and Cr is mainly due to the production of insoluble salts by metals sorption at higher pH [35,36]. The addition of lime or Na_2_S increases the pH of the system which in turn might help reduce the solubility with enhanced sorption and/or precipitation [37,38]. With the application of lime, the chances of the formation and precipitation of insoluble and moderately stable carbonate and hydroxide salts of the metal cations increase in higher pH (within 7–9). This noticeably immobilizes the bio-available fraction of HMs and reduced their accumulation in the system [39,40,41]. On the other side, the application of Na_2_S performs this job in two mechanistic ways: first, by forming sodium hydroxide (NaOH) and hydrogen sulphide (H_2_S) in the system which immobilizes HMs as metal sulphide ensuing the reduction with H_2_S followed by precipitation with NaOH [42], and second, by producing hydroxides during the dissolution process which in turn form metal hydroxide and increases pH. This is in the line of work of Lewis [43] who detected such a mechanism while working with cadmium. Ammonium-based fertilizers like DAP can potentially acidify the system, because ammonium is readily oxidized to nitrate in high soil pH, thereby releasing protons [44,45]. Phosphorus in the system with the application of DAP might have been able to form Pb/Cd/Ni/Cr-phosphate insoluble salts that minimize the availability of the respective HMs in the system [3,16]. Soil organic matter specifically humic acid has been of particular interest in this study because of the tendency of transition metal cations to form stable and strong complexes with organic ligands [46,47]. Thus, the incorporation of various ameliorants in the treatments helped to immobilize the free ions of HMs by complexation which in turn reduced its bio-availability in the system [48,49]. Contrarily, A1T0, A2T0, and A3T0 due to lack of ameliorant recorded the opposite trend with higher HMs accumulation.

Assessment of different ameliorants in reducing bio-availability of HMs showed that the treatments containing lime performed better (~41% higher decrease in bio-availability of Pb, Cd, Ni, and Cr) over others in our experiment (Figure 2). Overall comparison of different fly ash-soil-ameliorant combinations in maintaining less accumulation of bio-available HMs showed the superiority of A1T1 followed by A2T1 (Figure 4). Differences in the bio-availability of HMs among different treatment combinations could be attributed to the sorption capacity of the surface of the ameliorants with concomitant insolubility of the metal salts. The stability and insolubility of the carbonate and hydroxide salts are higher than that of the sulphate salts of HMs produced on the application of lime and Na_2_S respectively [50]. Na_2_S application also significantly increases electrical conductivity, salt pressure, and dispersive action in soils due to increased sodium concentrations [15,51]. Hence, Na_2_S may deteriorate the physical properties of base materials and reveal their unsuitability in our conditions. On the other hand, DAP, though mostly preferred, is easily available fertilizer input to farmers, but potentially acidifies the soil systems [52]. This ultimately affects the solubility of the metal salts and proved its poor efficiency in reducing metal bioavailability. Accordingly, lime performed better in reducing bio-available HMs as compared to DAP and Na_2_S. Furthermore, on the application of lime, Pb, Cd, Ni, and Cr may be physically entrapped within a solid matrix resulting from the pozzolanic reactions between lime and fly ash and leads to lower accumulation of such elements in the system [15]. Calcium aluminate hydrate and calcium silicate hydrate gels also have formed here due to the acid-base reaction of calcium from lime with alumina and silica in fly ash-soil combination [53,54]. Formation of these cementitious products adsorb, exchange, or encapsulate metals, fill the soil pores, and finally decrease HMs solubility as well as their bio-availability in the system [15,55]. On the contrary, humic acid could not act well due to the initial high pH of the system which possibly hampered the stability and insolubility of humic acid-HM complexes [56,57]. Additionally, adsorption or complexation of HMs induced by humic acid application also may be revived and/or remobilized into soils with the microbial degradation of humic matter over time [58,59]. The superiority of lime as an ameliorating agent in this regard was attested by many researchers for HMs [60] smelter-ash contaminated soils [61] as well as for fly ash-sewage sludge [62,63] soils. The efficacy of lime (at the rate of 5% and 10%) in reducing the bio-availability of Cd (71–99%) and Pb (61–88%) in an incubation study with metal contaminated soils was also reported by Mahar et al. (2018). This finding was further supported by the results of the variation in DTPA extractable HM to total- HM ratios (Figure 3) which showed a higher decrease in bio-available Pb, Cd, Ni, and Cr contents occurred in the limed treatments (~70% higher decrease over others) and A1T1 followed by A2T1 recoded the best performance. The strong correlation between bio-available metal to the ratio of metal bioavailability to total metal suggests that the changes in the reduction of the ratio are influenced by the bio-available fraction of metals which is subject to immobilization by different ameliorants (Table 4).

The potentiality of different treatment combinations in reducing the bio-availability of HMs was further assessed with PCA results (Table 4, Figure 5). The highest principal component score of A1T1 (2.32, Table 4) followed by A2T1 (2.13) in PC1 (explained 73%) indicates its significant performance in reducing bio-available HMs over other variables. Further, PCA-biplot depicted the presentation of different treatment combinations in reducing bio-availability of HMs where again A1T1 considered as the best while A3T0, the poorest (Figure 5). This finding may be attributed to the potentiality of lime in reducing the bio-availability of HMs as compared to other ameliorating agents. However, a fly ash-soil-ameliorant combination, to be qualified as the most suitable treatment in reducing bio-availability of HMs, must exhibit a satisfactory decline in HM accumulation from its initial in one side, and acts well with minimum risk to the environment, on the other. In this context, two useful indices, namely, CF and ERF were computed for different treatment combinations [64]. A1T1 attained the safest limit of CF for Ni, and Cr (CF < 1) and low to moderate levels for Pb, and Cd. Considering ERF, a similar trend was recorded like CF where A1T1 showed a low ERF level for all HMs (Figure 6). Again, the finding suggests that lime is the safest ameliorant while curbing the metal bioavailability in different fly ash soil combinations (Figure 2, Figure 3, Figure 4 and Figure 5).

Despite the well-tested ability of chemical amendments to reduce metal bioavailability, little attention has been paid towards assessing microbial endpoints under the influence of different treatments administered to contaminated environments. In this context, two highly sensitive bio-indicators, namely, MBC and dehydrogenase activity were assessed to detect the impact of treatments of soil microorganisms and their vital activity in the present investigation. Improvement of MBC load and dehydrogenase activity with the decreasing rate of fly ash suggests a lower accumulation of reactive metals inhibiting microbial growth and activities [65,66]. Significant improvement in MBC and dehydrogenase activity under the influence of different ameliorants in conjunction with different fly ash soil mixtures (A1 and A2) over sole fly ash (A3) is due to the attainment of low to moderate environmental risk factor (ERF) by lowering deleterious bio-available HMs. Humic acid sustained significantly the highest MBC and dehydrogenase activity over others as it supplies carbon to the microbes to facilitate their growth as well as for energy to perform different enzymatic activities [67]. Lime-treated fly ash soil mixtures proved their efficacy next to humic acid to harbor more microbes as reflected in MBC and also facilitate congenial ecological conditions for performing enzyme activity [68,69]. Accordingly, A1T4 followed by A1T1 performed the best.

Overall the study thus established the superiority of the A1T1 combination (50% fly ash + 50% soil+ lime) in reducing bio-availability of HMs along with minimum ecological risk and improved microbial habitat.

## 5. Conclusions

Fly ash as soil ameliorant has been a topic of scientific research for a long time and its performance is well documented as per bibliographic antecedent. Rejuvenating fly ash at dumping sites for agricultural production by arresting metal mobility through chemical ameliorants applications is a new approach as per our knowledge. Accordingly, to perform this task, fifteen different fly ash-soil-ameliorant combinations were studied under 60-days incubation to evaluate their effectiveness in reducing the bio-availability of heavy metals. Fly ash-soil (50% basis) + lime, i.e., A1T1 has been worked out from the current experimental finding as to the best approach in this direction. In addition, a low environmental risk factor for Pb, Cd, Ni, and Cr, as well as congenial microbial ecology for supporting higher MBC load and dehydrogenase activity were achieved by the conjoint application of lime and 50:50 fly ash-soil mixture. To discern its potentiality, the applicability of this finding should be tested in situ in the ash pond areas of a power plant by growing root, leafy, and fruit vegetables for understanding the metal profile in different vegetable crops in diverse agro-ecological conditions. More so, metal accumulation in crop plants is to be tested to comply with the World Health Organization guideline for the permissible limit of metals in the edible portion of crop plants.

## Figures and Tables

**Table 1:** Pearsons’ correlation matrix between bio-available to total heavy metal (HM) ratios and different forms of HMs.

HM Fractions	Bio-Available to Total HM Ratios			
	Pb	Cd	Ni	Cr
Bio-available	0.76 **	0.30	0.97 **	0.86 **
Total	0.46	0.36	0.70 **	0.35

** indicate significant correlation at 0.01 levels of confidence.

**Table 2:** Loadings (Eigen vectors) of different fly ash-soil-ameliorant combinations for reducing bio-availability of heavy metals by principal component analysis (PCA).

Fly Ash-Soil-Ameliorant Combinations	PC1	PC2	PC3
A1T0	−1.705	−0.676	0.749
A1T1	2.328	−0.478	−0.142
A1T2	0.608	−0.909	0.292
A1T3	1.634	0.111	−0.209
A1T4	−0.276	−0.886	−0.312
A2T0	−2.705	−0.711	0.225
A2T1	2.132	−0.326	0.238
A2T2	1.041	−1.041	0.359
A2T3	1.418	1.195	0.292
A2T4	−0.423	−0.095	−1.364
A3T0	−3.622	0.644	0.024
A3T1	0.732	0.892	0.087
A3T2	−0.452	0.914	−0.256
A3T3	0.0749	1.600	0.398
A3T4	−0.784	−0.231	−0.383
Standard deviation	1.71	0.84	0.49
Proportion of Variance (%)	73.36	18.05	6.02
Cumulative Proportion (%)	73.36	91.41	97.43

A1: fly ash (50%) + soil (50%), A2: fly ash (75%) + soil (25%), A3: fly ash (100%); T0: without ameliorants, T1: lime at 5 Mg ha^−1^, T2: sodium sulfide at 2 Mg ha^−1^, T3: di-ammonium phosphate at 0.5 Mg ha^−1^, T4: humic acid at 4 Mg ha^−1^; PC: principal component.

**Table 3:** Microbial biomass carbon (MBC; µg g^−1^) and dehydrogenase activity (µg TPF g^−1^ h^−1^) under different fly ash-soil-ameliorant combinations.

Fly Ash-Soil-Ameliorant Combinations	MBC	Dehydrogenase
A1T0	34.34 ^e^ ± 1.2	4.35 ^f^ ± 1.26
A1T1	65.84 ^b^ ± 0.81	11.92 ^b^ ± 1.16
A1T2	55.45 ^c^ ± 0.81	7.64 ^d^ ± 0.81
A1T3	56.82 ^c^ ± 0.21	8.80 ^c^ ± 0.65
A1T4	79.53 ^a^ ± 0.99	13.8 ^a^ ± 1.24
A2T0	18.39 ^g^ ±1.63	1.77 ^g^ ± 0.31
A2T1	31.24 ^f^ ± 1.65	5.07 ^e^ ± 0.11
A2T2	33.89 ^ef^ ± 1.85	5.06 ^e^ ± 0.60
A2T3	33.86 ^ef^ ± 1.34	5.11 ^e^ ± 0.77
A2T4	45.09 ^d^ ± 0.66	7.69 ^d^ ± 1.10
A3T0	4.88 ^i^ ± 0.43	0.47 ^h^ ± 0.11
A3T1	4.84 ^i^ ± 0.99	0.47 ^h^ ± 0.07
A3T2	6.78 ^i^ ± 0.94	0.50 ^h^ ± 0.01
A3T3	4.68 ^i^ ± 1.23	0.29 ^h^ ± 0.02
A3T4	11.63 ^h^ ± 2.22	0.63 ^h^ ± 0.12

Values followed by different lowercase letters are significantly different Tukey’s test (*p* = 0.05) for heavy metal; A1: fly ash (50%) + soil (50%), A2: fly ash (75%) + soil (25%), A3: fly ash (100%); T0: without ameliorants, T1: lime at 5 Mg ha^−1^, T2: sodium sulfide at 2 Mg ha^−1^, T3: di-ammonium phosphate at 0.5 Mg ha^−1^, T4: humic acid at 4 Mg ha^−1.^

**Table 4:** Physio-chemical and biological properties of experimental materials.

Parameters	Fly Ash	Soil	Referenced Methods
Physical properties			
Sand (%)	56.66	29.47	Bouyoucos [20]
Silt (%)	38.45	52.20	
Clay (%)	4.32	18.33	
Textural Class	Sandy silty loam	Silty loam	
Bulk density (g cm^−3^)	1.01	1.35	
Physio-chemical and chemical properties			
pH (1:2.5, H_2_O)	7.91	7.01	Jackson [21]
EC (dsm^−1^)	0.41	0.21	
Organic C (%)	0.004	0.61	Walkley and Black [22]
Available N (mg kg^−1^)	-	92.31	Subbai and Asija [23]
Olsen-P (mgkg^−1^)	46.21	16.38	Olsen et al. [24]
NH_4_OAc-K	85.32	80.51	Hanway and Heidel [25]
Fe (mgkg^−1^)	12.11	7.45	Lindsay and Norvell [26]
Mn (mgkg^−1^)	9.15	14.21	
Zn (mgkg^−1^)	1.98	1.08	
Cu (mgkg^−1^)	2.49	1.66	
Biological properties			
MBC (µg g^−1^)	15.21	188.61	Vance et al. [27]
Dehydrogenase (µg TPF g^−1^ h^−1^)	2.14	19.21	Klein et al. [28]

**Table 5:** Fly ash-soil-ameliorants combinations were used in this experiment.

Different Combinations of Treatments	Notations
50% fly ash + 50% soil + no ameliorant	A1T0
50% fly ash + 50% soil + lime at 5 Mg ha^−1^	A1T1
50% fly ash + 50% soil + sodium sulphide at 2 Mg ha^−1^	A1T2
50% fly ash + 50% soil + di-ammonium phosphate at 0.5 Mg ha^−1^	A1T3
50% fly ash + 50% soil + humic acid at 4 Mg ha^−1^	A1T4
75% fly ash + 25% soil + no ameliorant	A2T0
75% fly ash + 25% soil + lime at 5 Mg ha^−1^	A2T1
75% fly ash + 25% soil + sodium sulphide at 2 Mg ha^−1^	A2T2
75% fly ash + 25% soil + di-ammonium phosphate at 0.5 Mg ha^−1^	A2T3
75% fly ash + 25% soil + humic acid at 4 Mg ha^−1^	A2T4
100% fly ash + no ameliorant	A3T0
100% fly ash + lime at 5 Mg ha^−1^	A3T1
100% fly ash + sodium sulphide at 2 Mg ha^−1^	A3T2
100% fly ash + di-ammonium phosphate at 0.5 Mg ha^−1^	A3T3
100% fly ash + humic acid at 4 Mg ha^−1^	A3T4

## Data Availability

The datasets used and/or analyzed during the current study are available from the first author on reasonable request.

## Sample Availability

Samples of the compounds are not available from the authors.

## References

[1] Belyaeva O.N., Haynes R.J. (2012). Comparison of the effects of conventional organic amendments and biochar on the chemical, physical and microbial properties of coal fly ash as a plant growth medium. Environ. Earth Sci..

[2] Tripathi R.C., Jha S.K., Ram L.C. (2016). Impact of heavy metals in Indian fly ashes on its application as soil ameliorant. Energy Sources Part A.

[3] Basta N.T., McGowen S.L. (2004). Evaluation of chemical immobilization treatments for reducing heavy metal transport in a smelter-contaminated soil. Environ. Pollut..

[4] Roy M., Roychowdhury R., Mukherjee P. (2018). Remediation of fly ash dumpsites through bioenergy crop plantation and generation: A review. Pedosphere.

[5] Bryan A.L., Hopkins W.A., Parikh J.H., Jackson B.P., Unrine J.M. (2012). Coal fly ash basins as an attractive nuisance to birds: Parental provisioning exposes nestlings to harmful trace elements. Environ. Pollut..

[6] Guleria A., Chakma S. (2021). Probabilistic human health risk assessment of groundwater contamination due to metal leaching: A case study of Indian dumping sites. Hum. Ecol. Risk Assess..

[7] Sett R. (2017). Flyash: Characteristics, problems and possible utilization. Adv. Appl. Sci. Res..

[8] Jambhulkar H.P., Shaikh S.M.S., Kumar M.S. (2018). Fly ash toxicity, emerging issues and possible implications for its exploitation in agriculture; Indian scenario: A review. Chemosphere.

[9] Basu M., Pande M., Bhadoria P.B.S., Mahapatra S.C. (2009). Potential fly-ash utilization in agriculture: A global review. Prog. Nat. Sci..

[10] Sahu G., Bag A.G., Chatterjee N., Mukherjee A. (2017). Potential use of flyash in agriculture: A way to improve soil health. J. Pharmacogn. Phytochem..

[11] Pandey V.C., Singh K., Singh R.P., Singh B. (2012). Naturally growing *Saccharum munja* L. on the fly ash lagoons: A potential ecological engineer for the revegetation and stabilization. Ecol. Eng..

[12] Pandey V.C., Singh N. (2014). Fast green capping on coal fly ash basins through ecological engineering. Ecol. Eng..

[13] Rai U.N., Pandey K., Sinha S., Singh A., Saxena R., Gupta D.K. (2004). Revegetating fly ash landfills with *Prosopis juliflora* L.: Impact of different amendments and Rhizobium inoculation. Environ. Int..

[14] Gupta A.K., Dwivedi S., Sinha S., Tripathi R.D., Rai U.N., Singh S.N. (2007). Metal accumulation and growth performance of *Phaseolus vulgaris* grown in fly ash amended soil. Bioresour. Technol..

[15] Mahar A., Wang P., Ali A., Lahori A.H., Awasthi M.K., Wang Z., Zhang Z. (2018). (Im) mobilization of soil heavy metals using CaO, FA, sulfur, and Na_2_S: A 1-year incubation study. Int. J. Environ. Sci. Technol..

[16] Zhou C., Yuan H., Ning C., Li S., Xia Z., Zhu M., Ma Q., Yu W. (2020). Evaluation of different types and amounts of amendments on soil Cd immobilization and its uptake to wheat. Environ. Manag..

[17] Mukherjee S., Saha N., Sarkar B., Sengupta S., Ghosh S., Dey P. (2021). Assessing Methods for Estimating Potentially Mineralisable Nitrogen Under Organic Production System in New Alluvial Soils of Lower Gangetic Plain. Soil Sci. Plant Nutr..

[18] Soil Survey Staff (2003). Keys to Soil Taxonomy.

[19] Page A.L., Miller R.H., Keeney D.R. (1982). Methods of soil analysis, part 2. Chem. Microbiol. Prop..

[20] Bouyoucos G.J. (1962). Hydrometer method improved for making particle size analyses of soils 1. Agron J..

[21] Jackson M.L. (2005). Soil Chemical Analysis: Advanced Course.

[22] Walkley A., Black I.A. (1934). An examination of the Degtjareff method for determining soil organic matter, and a proposed modification of the chromic acid titration method. Soil Sci..

[23] Subbaiah V.V., Asija G.K. (1956). A rapid procedure for utilization of available nitrogen in soil. Curr. Sci..

[24] Olsen S.R. (1954). Estimation of Available Phosphorus in Soils by Extraction with Sodium Bicarbonate.

[25] Hanway J.J., Heidal H. (1952). Soil analysis methods as used in Iowa State College Soil Testing Laboratory. Iowa State Coll. Agric. Bull..

[26] Lindsay W.L., Norvell W.A. (1978). Development of a DTPA soil test for zinc, iron, manganese, and copper. Soil Sci. Soc. Am. J..

[27] Vance E.D., Brookes P.C., Jenkinson D.S. (1987). An extraction method for measuring soil microbial biomass C. Soil Biol. Biochem..

[28] Klein D.A., Loh T.C., Goulding R.L. (1971). A rapid procedure to evaluate the dehydrogenase activity of soils low in organic matter. Soil Biol. Biochem..

[29] Allen S.E., Grimshaw H.M., Rowland A.P., Moore P.D., Chapman S.B. (1986). Chemical analysis. Methods in Plant Ecology.

[30] Hakanson L. (1980). An ecological risk index for aquatic pollution control. A sedimentological approach. Water Res..

[31] Shapiro S.S., Wilk M.B. (1965). An analysis of variance test for normality (complete samples). Biometrika.

[32] Gomez K.A., Gomez A.A. (1984). Statistical Procedures for Agricultural Research.

[33] Enderlein G., Hawkins D.M. (2010). Identification of Outliers. Chapman and Hall, London–New York 1980. Biom. J..

[34] Pandey S.K., Bhattacharya T. (2019). Mobility, ecological risk and change in surface morphology during sequential chemical extraction of heavy metals in fly ash: A case study. Environ. Technol. Innov..

[35] Yang Z., Jing F., Chen X., Liu W., Guo B., Lin G., Huang R., Liu W. (2018). Spatial distribution and sources of seven available heavy metals in the paddy soil of red region in Hunan Province of China. Environ. Monit. Assess..

[36] Zhong X., Chen Z., Li Y., Ding K., Liu W., Liu Y., Yuan Y., Zhang M., Baker A.J., Yang W. (2020). Factors influencing heavy metal availability and risk assessment of soils at typical metal mines in Eastern China. J. Hazard. Mater..

[37] González-Alcaraz M.N., Conesa H.M., Álvarez-Rogel J. (2013). Phytomanagement of strongly acidic, saline eutrophic wetlands polluted by mine wastes: The influence of liming and Sarcocornia fruticosa on metals mobility. Chemosphere.

[38] Zhang X., Lou X., Zhang H., Ren W., Tang M. (2020). Effects of sodium sulfide application on the growth of Robinia pseudoacacia, heavy metal immobilization, and soil microbial activity in Pb–Zn polluted soil. Ecotoxicol. Environ. Saf..

[39] Vítková M., Ettler V., Šebek O., Mihaljevič M., Grygar T., Rohovec J. (2009). The pH-dependent leaching of inorganic contaminants from secondary lead smelter fly ash. J. Hazard. Mater..

[40] Ruttens A., Adriaensen K., Meers E., De Vocht A., Geebelen W., Carleer R., Mench M., Vangronsveld J. (2010). Long-term sustainability of metal immobilization by soil amendments: Cyclonic ashes versus lime addition. Environ. Pollut..

[41] Yuan X., Leng L., Huang H., Chen X., Wang H., Xiao Z., Zhai Y., Chen H., Zeng G. (2015). Speciation and environmental risk assessment of heavy metal in bio-oil from liquefaction/pyrolysis of sewage sludge. Chemosphere.

[42] Prokkola H., Nurmesniemi E.T., Lassi U. (2020). Removal of Metals by Sulphide Precipitation Using Na_2_S and HS—Solution. ChemEngineering.

[43] Lewis A.E. (2010). Review of metal sulphide precipitation. Hydrometallurgy.

[44] Liu J., Su Y., Li Q., Yue Q., Gao B. (2013). Preparation of wheat straw based superabsorbent resins and their applications as adsorbents for ammonium and phosphate removal. Bioresour. Technol..

[45] Goulding K.W.T. (2016). Soil acidification and the importance of liming agricultural soils with particular reference to the United Kingdom. Soil Use Manag..

[46] Yip T.C., Yan D.Y., Yui M.M., Tsang D.C., Lo I.M. (2010). Heavy metal extraction from an artificially contaminated sandy soil under EDDS deficiency: Significance of humic acid and chelant mixture. Chemosphere.

[47] Xu Y. (2017). Stabilization of heavy metal-contaminated sediment with a chelator and humic acid mixture. Water Air Soil Pollut..

[48] Skłodowski P., Maciejewska A., Kwiatkowska J. (2006). The effect of organic matter from brown coal on bioavailability of heavy metals in contaminated soils. Soil and Water Pollution Monitoring, Protection and Remediation.

[49] Wuana R.A., Okieimen F.E. (2011). Heavy metals in contaminated soils: A review of sources, chemistry, risks and best available strategies for remediation. Int. Sch. Res. Notices.

[50] Pachana K., Wattanakornsiri A., Nanuam J. (2010). Heavy metal transport and fate in the environmental compartments. Int. J. Sci..

[51] Zhang H., Tian Y., Guan B., Zhou D., Sun Z., Baskin C.C. (2018). The best salt solution parameter to describe seed/seedling responses to saline and sodic salts. Plant Soil.

[52] Sarwar N., Malhi S.S., Zia M.H., Naeem A., Bibi S., Farid G. (2010). Role of mineral nutrition in minimizing cadmium accumulation by plants. J. Sci. Food Agric..

[53] Hale B., Evans L., Lambert R. (2012). Effects of cement or lime on Cd, Co, Cu, Ni, Pb, Sb and Zn mobility in field-contaminated and aged soils. J. Hazard. Mater..

[54] Shaheen S.M., Rinklebe J. (2015). Impact of emerging and low cost alternative amendments on the (im) mobilization and phytoavailability of Cd and Pb in a contaminated floodplain soil. Ecol. Eng..

[55] Singh T.S., Pant K.K. (2006). Solidification/stabilization of arsenic containing solid wastes using portland cement, fly ash and polymeric materials. J. Hazard. Mater..

[56] Wu S., Li R., Peng S., Liu Q., Zhu X. (2017). Effect of humic acid on transformation of soil heavy metals. IOP Conf. Ser. Mater. Sci. Eng..

[57] Khan R.U., Khan M.Z., Khan A., Saba S., Hussain F., Jan I.U. (2018). Effect of humic acid on growth and crop nutrient status of wheat on two different soils. J. Plant Nutr..

[58] Evangelou M.W., Daghan H., Schaeffer A. (2004). The influence of humic acids on the phytoextraction of cadmium from soil. Chemosphere.

[59] Park J.H., Choppala G.K., Bolan N.S., Chung J.W., Chuasavathi T. (2011). Biochar reduces the bioavailability and phytotoxicity of heavy metals. Plant Soil.

[60] Castaldi P., Santona L., Melis P. (2005). Heavy metal immobilization by chemical amendments in a polluted soil and influence on white lupin growth. Chemosphere.

[61] Hong C.O., Gutierrez J., Yun S.W., Lee Y.B., Yu C., Kim P.J. (2009). Heavy metal contamination of arable soil and corn plant in the vicinity of a zinc smelting factory and stabilization by liming. Arch. Environ. Contam. Toxicol..

[62] Lagerwerff J.V., Biersdorf G.T., Milberg R.P., Brower D.L. (1977). Effects of Incubation and Liming on Yield and Heavy Metal Uptake by Rye from Sewage-Sludged Soil. J. Environ. Qual..

[63] Hooda P.S., Alloway B.J. (1996). The effect of liming on heavy metal concentrations in wheat, carrots and spinach grown on previously sludge-applied soils. J. Agric. Sci..

[64] Silva H.F., Silva N.F., Oliveira C.M., Matos M.J. (2021). Heavy Metals Contamination of Urban Soils—A Decade Study in the City of Lisbon, Portugal. Soil Syst..

[65] Nayak A.K., Kumar A., Raja R., Rao K.S., Mohanty S., Shahid M., Tripathy R., Panda B.B., Bhattacharyya P. (2014). Fly ash addition affects microbial biomass and carbon mineralization in agricultural soils. Bull. Environ. Contam. Toxicol..

[66] Ghosh R.K., Singh N., Singh S.B. (2016). Effect of fly ash amendment on metolachlor and atrazine degradation and microbial activity in two soils. Environ. Monit. Assess..

[67] Doğan K., Sarıoğlu A., Coşkan A. (2016). Contribution of Green Manure, Rhizobium and Humic + Fulvic Acid on Recovering Soil Biologic Activity of Olive Mill Wastewater Contaminated Soil. Sci. Pap. Ser. A Agron..

[68] Xue D., Huang X., Yao H., Huang C. (2010). Effect of lime application on microbial community in acidic tea orchard soils in comparison with those in wasteland and forest soils. J. Environ. Sci..

[69] Inagaki T.M., de Moraes Sá J.C., Caires E.F., Gonçalves D.R.P. (2017). Why does carbon increase in highly weathered soil under no-till upon lime and gypsum use?. Sci. Total Environ..

